# What Do Students’ Questionnaire Responses Tell Us about Their Language around Person-Centred Care? An Exploratory Sentiment Analysis

**DOI:** 10.3390/healthcare11172458

**Published:** 2023-09-03

**Authors:** Helen Wood, Gabrielle Brand, Rhonda Clifford, Sinead Kado, Kenneth Lee, Liza Seubert

**Affiliations:** 1 School of Allied Health, The University of Western Australia, Perth 6009, Australia; rhonda.clifford@uwa.edu.au (R.C.); sinead.kado@research.uwa.edu.au (S.K.); kenneth.lee@uwa.edu.au (K.L.); liza.seubert@uwa.edu.au (L.S.); 2 School of Nursing & Midwifery, Monash University, Melbourne 3004, Australia; gabrielle.brand@monash.edu

**Keywords:** health care professionals, social care professionals, health education, person-centred care

## Abstract

There is a global movement for health and social care to be person-centred: supporting people’s active participation when making health decisions and considering their opinions, beliefs, and needs. The World Health Organization recommend the inclusion of person-centred care in health and social care provision. This research aimed to explore Australian health and social care profession students’ language around person-centred care. Final-year health and social care professions students, attending one of two Australian universities, participated in an online questionnaire. Responses were analysed and themed to an existing person-centred care framework, then a sentiment analysis was applied to each response. Of the responses collected from 90 students, 235 statements were linked to the four core values of the person-centred care framework: cultivating communication (44%); respectful and compassionate care (35%); engaging patients in managing their care (20%); and integration of care (<1%). Within these, 24 statements were positively aligned (10%); 100 statements were neutral (43%); and 111 statements contained negative sentiments (47%). Almost half of the responses were not aligned with the core values of person-centred care. This suggests that many of the final-year students are not yet conceptualizing care using a person-centred approach.

## 1. Introduction

To provide care to individuals in a holistic and compassionate manner, health and social care professionals must not only have relevant knowledge and skills; they must also deliver care using a person-centred approach [1]. Person-centred care focuses on the person as a whole, taking into account their opinions, beliefs, and needs, rather than primarily seeing them as a passive patient or medical condition [2]. Approaching care from a person-centred perspective allows the health or social care professional to support the person’s active participation in their health decisions, care, and treatment [2,3], working towards the goal of achieving a meaningful—rather than functional—life [3]. The benefits of a person-centred care approach extend further, with the potential to improve people’s access to care, health care satisfaction, health outcomes, and individual health literacy [4,5,6,7].

The move towards embedding person-centredness in care has gained momentum in recent years. The World Health Organization developed a global strategy to integrate person-centred care into all health and social services [4], and the Picker Institute Europe published a policy briefing that showed evidence of legislative frameworks within European countries that seek to improve the quality of person-centred care [8]. Within Australia, person-centred care is becoming increasingly embedded into the professional practice standards of health and social care professionals. As an example, the Pharmaceutical Society of Australia places person-centred care at the heart of 15 of the 16 most recently published professional standards [9], whereas the previous version only mentions aspects of person-centred care, and far less prominently [10]. Similarly, Australian registered nurses’ professional standards primarily describe the nursing practice as being person-centred and evidence-based, demonstrated by the provision of care that respects, protects, and empowers individuals [11].

In 2011, the Australian Commission on Safety and Quality in Healthcare (ACSQHC)(Sydney, Australia) published a set of recommendations for actions to improve the provision and quality of person-centred care [12]. One such system-oriented recommendation—designed to improve person-centred care across the entire healthcare system—was to include person-centred care as a component of Australian health professions curricula. Subsequent research into the effectiveness of person-centred care education in Australia is limited; to date, self-reported satisfaction with the health professions curriculum is the most frequently used metric [13,14]. While the results have shown high levels of satisfaction, evaluation needs to extend beyond self-reported satisfaction and alternative methodologies using a different lens should be used to further explore the topic.

Many of the core values that health and social care professionals need when providing person-centred care—such as clear communication and cultural capability—are also essential when caring for someone in a way that responds to their health literacy needs [5,12]. Health literacy, described as an individual’s ability to access, understand, appraise, and use relevant health information to improve their health [15], is shaped not only by individual characteristics but also by health and social care professionals [12]. A recently published study explored health and social care professions students’ health literacy knowledge [16]; whilst analysing the data, researchers also noticed that many responses gave fresh insights into students’ attitudes towards person-centred care. Considering the identified need to further explore the inclusion of person-centredness in health and social care professions’ curricula, a secondary data analysis was conducted using a person-centred care lens.

Aim: Using sentiment analysis, the aim of this study was to explore health and social care professions students’ language around providing person-centred care.

## 2. Materials and Methods

### 2.1. Overview

A secondary analysis was conducted on the raw data from a previous survey [16], which explored students’ health literacy knowledge across different health and social care profession disciplines. An anonymous web-based platform was used to survey final-year health and social care professions students enrolled in one of two Australian universities. Approval for this research was granted by the Human Research Ethics Committee at the University of Western Australia (RA/4/20/5960). The Checklist for Reporting Results of Internet E-Surveys (CHERRIES) was adhered to for transparent reporting (Supplementary Material S1) [17].

### 2.2. Questionnaire Development

The questionnaire was developed by researchers with expertise in health literacy research (H.W., G.B., and K.L.). There were two components of the questionnaire that were included in this secondary analysis: the first section collected demographic information, and the second section contained a definition of the term ‘health literacy’ (“Health literacy is the degree to which individuals have the capacity to obtain, process, and understand basic health information and services needed to make appropriate health decisions” [18]) with three open-text boxes asking students to describe three signs or behaviours that indicate a person may have inadequate health literacy (Supplementary Material S2). The questionnaire was uploaded to Qualtrics, a web-based survey platform, along with a two-page Participant Information Form and consent statement. A forced response was applied for the consent statement; students could leave any other question unanswered. Prior to dissemination, five health professions students piloted the questionnaire to ensure correct formatting on different devices and that questions were easily understood. No changes were recommended, and data collected during piloting was not included in the analysis. A copy of the complete survey can be obtained from the original study [16].

### 2.3. Participants and Recruitment

Final-year students enrolled in the following degree programmes in 2021 were invited to participate: Dental Medicine; Nursing; Pharmacy; Podiatric Medicine; and Social Work and Social Policy. Students were offered the opportunity to enter a draw to win an AUD 25 e-gift card but their contact information was viewable only by one research member (H.W.) and not linked to survey responses. The Learning Management System was used to broadcast recruitment announcements directly to students, which was viewable via the software or email. The survey was made available for four weeks, and a reminder was sent to all eligible participants one week prior to the closing date.

### 2.4. Analysis and Reporting

After data immersion, two researchers (H.W. and S.K.), a pharmacy educator and health professions educator, respectively, independently analysed the data in two stages. The first stage involved a content analysis using a pre-existing person-centred care framework, with the second stage being a sentiment analysis of the meaning within the text. Although the questions students answered were related to health literacy rather than providing person-centred care, this sub-analysis did not evaluate responses using a health literacy perspective.

**Figure 1:**
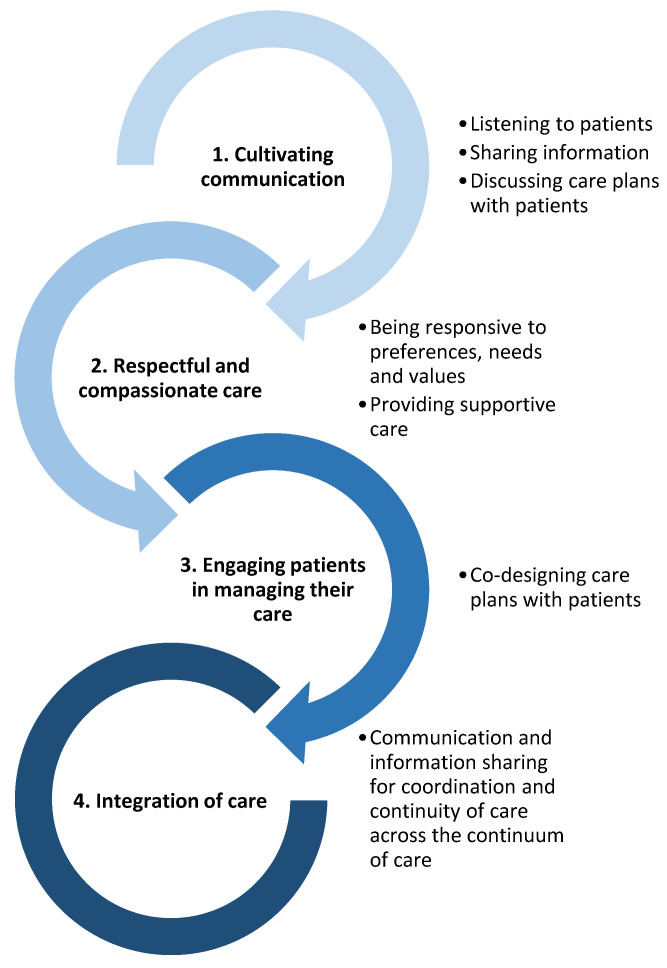
Themes and sub-themes used during data analysis. Numbers 1–4 are the themes; dot points beside each theme are the sub-themes. Themes and sub-themes were based on a framework developed by Santana et al. [19].

Santana et al. developed a framework to guide the provision of person-centred care, which formed the basis of the codebook used in the content analysis [19]. The framework, used to guide codebook development in other health professional-related studies [20,21], was conceptualized using patients’ and carers’ lived experiences, and supplemented with additional research on person-centred care [19]. Of the three domains in Santana et al.’s framework, only the “Patient-healthcare provider” domain was used because the other two domains were not relevant to our research aim. The first omitted domain, Structure, explores the health system from an organisation level (where practising health and social care professionals often have limited influence). The second omitted domain, Outcome, focuses on access to care (which, again, is more relevant to an organisation level) and patient-reported outcomes (such as quality of life or recovery, which are patient-centric). The patient-healthcare provider domain is comprised of four core values, which were used as themes in our codebook. Each core value had between one to three components, which were used as sub-themes. Santana et al. developed two to five descriptors per component; these were used to supplement the codebook development. The person-centred care themes and sub-themes derived from Santana et al.’s framework are shown in Figure 1.

A response was determined to relate to ‘Cultivating communication’ when the student described how a person may struggle to be heard, receive information, or engage in discussion with them as a health or social care professional (example: “No knowledge about their condition—ask them a question in regards to their condition but they can’t tell you anything or limited information”). A response was counted as ‘Respectful and compassionate care’ when the student acknowledged an individual’s circumstances, which may need to be considered when providing person-centred care (example: “Poor numeracy and literacy skills”). Responses that aligned with ‘Engaging patients in managing their care’ were those where the student identified signs that a person may struggle to co-design and/or implement an achievable care plan (example: “Non-compliance with a treatment plan”). A response was coded as ‘Integration of care’ when the student described a person who may have difficulty following referral pathways (example: “Unwilling to see a doctor or go to hospital when referred”). The researchers agreed to deviate from Santana et al.’s given description of one sub-theme for the person-centred care codebook (‘gathering information through active listening’ became ‘gathering information’, as this still fit the sub-theme of ‘Listening to patients’).

After data were analysed according to the person-centred care framework, sentiment analysis was manually applied. Sentiment analyses have been successfully used when exploring health-related tweets [22,23,24], which are data of up to 280 characters in length—comparable to our survey response data. Sentiments were categorized as ‘positive’, ‘neutral’, or ‘negative’. A response was determined to be ‘positive’ when the student acknowledged a core value of person-centred care and demonstrated either a way to provide it or an attempt to understand the person’s perspective (example: “They don’t take medications because they don’t have a clear understanding of their diagnosis/no one has ever explained it to them”). A response was determined to be ‘neutral’ when the student only acknowledged an aspect of person-centred care (example: “Looking confused and cannot follow up during conversations”, which indicates that the student is aware that a shared understanding of information has not been reached, but the statement does not indicate that the student is ready to bridge the gap in knowledge). A ‘negative’ response was one where the statement did not align with person-centred language (examples: “Believer in complementary and alternative medicines”, which does not recognize or value the beliefs of different cultural groups; “[The person] asks questions about their health or treatment” does not demonstrate a willingness to reach a shared understanding) or the language used was negative and emotive (example: “Disobeying recommendations, e.g., medications administration”).

H.W. themed all responses using the updated theming process, and S.K. was allocated a different randomly selected 20% of responses to independently theme. The agreement was higher than 95%, with no further changes identified.

## 3. Results

### 3.1. Demographics

As reported in the previous study [16], 90 students submitted responses for inclusion. Participating students were more likely to be 23 years old (interquartile range 2), female (*n* = 61; 68%), and a pharmacy student (*n* = 31; 34%) (Table 1).

As each student was able to provide up to three answers, 270 responses were submitted. Of these, eight fields were left blank and a further 27 responses were excluded as they were irrelevant to the concept of person-centred care (example: “Not know who to access for certain conditions”). A total of 235 responses from 90 participants were included in the analysis.

### 3.2. Overview

Of the 235 relevant responses, 24 (10%) contained a positive sentiment, 100 (43%) were neutral and 111 (47%) contained a negative sentiment.

### 3.3. Cultivating Communication

**Figure 2:**
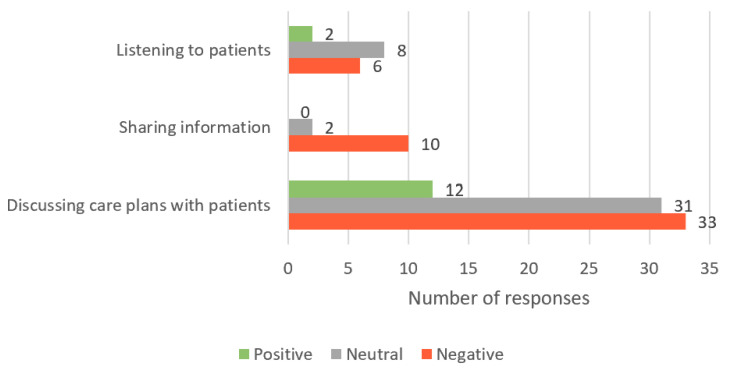
Number of student responses, which aligned to the ‘Cultivating communication’ core value of person-centred care; responses are grouped by both sub-theme and sentiment.

Cultivating communication reflects students’ ability to actively listen to people in order to identify their preferences, values and needs, share information with the person to reach a mutual understanding, and discuss care plans so the person is equipped to make an informed health decision. Of the 235 responses, 104 responses fit this theme. Overall, 14 (13%) responses contained positive sentiments (example: “Not understanding the purpose of the procedure/treatment (maybe just memorizing but not actually understanding)”), 41 (39%) responses were neutral (example: “Don’t know why they need to keep their diabetes under control”), and 49 (47%) responses indicated negative sentiments (example: “Aggressively believing misinformation, and hence engages in anti-social behaviour with the health professional”) (Figure 2).

### 3.4. Respectful and Compassionate Care

**Figure 3:**
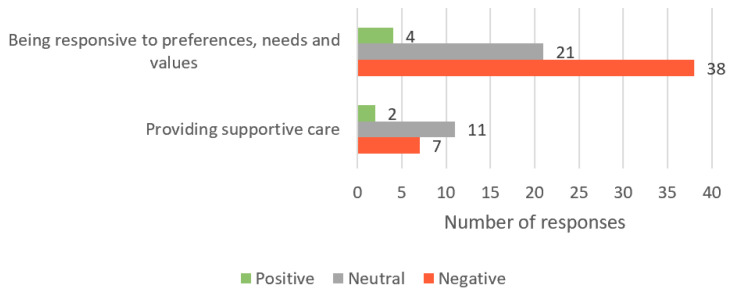
Number of student responses, which aligned to the ‘Respectful and compassionate care’ core value of person-centred care; responses are grouped by both sub-theme and sentiment.

Respectful and compassionate care reflects students’ ability to acknowledge the person as a core member of the health and/or social care team, respond to their preferences, values and needs, display empathy, and appropriately identify and respond to the person’s emotional needs. Of the 235 responses, 83 were included in this theme. Overall, 6 (7%) responses were determined to contain positive sentiments (example: “Language barrier—when trying to communicate to them they aren’t able to understand and comprehend what you are saying in English”), 32 (39%) were neutral (example: “Introverted demeanour when consulting a health professional”), and 45 (54%) contained negative sentiments (example: “When they do not scrutinize their own mislead beliefs”) (Figure 3).

### 3.5. Engaging Patients in Managing Their Care

**Figure 4:**
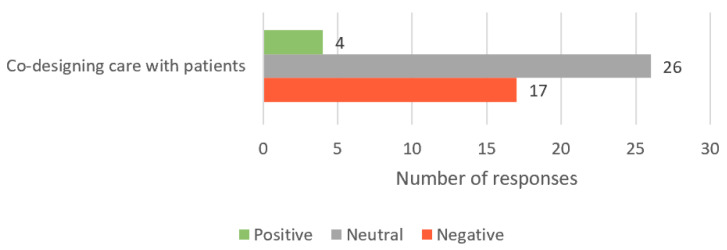
Number of student responses, which aligned to the ‘Engaging patients in managing their care’ core value of person-centred care; as this theme has only one sub-theme, responses are only grouped by sentiment.

Responses in this theme reflect the students’ ability to promote shared decision making, support the person to manage self-care and help them feel empowered and respected. Of the 235 responses, 47 fit this theme. Overall, 4 (9%) responses contained positive sentiments (example: “Gives their power completely to a professional rather than valuing their own thoughts and perspectives”), 26 (55%) responses were neutral (example: “Engaging in activities that could harm them and their condition”), and 17 (36%) indicated negative sentiments (example: “Ignorant about managing their own health”) (Figure 4).

### 3.6. Integration of Care

Integration of care refers to the student identifying the need for collaboration with or referral to other health and social care professionals and maintaining continuity of care via access to referral pathways, resources, or discharge communication. Of the 235 responses, one response was aligned with this theme; it contained a neutral sentiment (“Unwilling to see doctor or go to hospital when referred”).

## 4. Discussion

The use of a sentiment analysis allowed an interesting new perspective on health and social care professions students’ language around person-centred care when considering health literacy-related needs or challenges. By aligning responses to a person-centred care framework, we were able to identify that many students were not oriented towards person-centred care in three of the four core values: Cultivating communication; respectful and compassionate care; and engaging patients in managing their care. Despite person-centred care values being mandated as part of professional practice standards, this study demonstrates that the professional standards are not reflected in the language they are using.

Communication is an essential component of providing person-centred care; by listening, sharing information, and discussing care plans, health and social care professionals can help people understand in order to actively participate in their care [19]. Effective person-centred communication has numerous demonstrated benefits; higher satisfaction, information recall, health understanding, and adherence, as well as improved health outcomes, have been reported when communication is person-centred [6,7,19,25]. While there were a small number of positive sentiments, almost half of the students’ responses contained negative sentiments which were incongruent with cultivating communication. This could be an indication that, despite being in their final year of study, their ideas around communication are not aligned with a person-centred approach.

The importance of providing respectful and compassionate care, the second core value of providing person-centred care, cannot be overstated. When health and social care professionals understand the person’s unique perspective—including their values, beliefs, culture, and needs—it helps build a trusting relationship, leads to increased satisfaction with care, and supports treatment adherence and motivation [26,27,28,29,30,31]. Additionally, health and social care professionals can achieve similar outcomes by responding to people’s emotional cues, demonstrating empathy, and providing reassurance [26,30,32,33]. In this study, just over half of students’ responses displayed a negative sentiment and few responses were positively aligned, suggesting that they may not frame their health or social care in a respectful and compassionate way, potentially affecting their ability to provide person-centred care.

The third core value of person-centred care is the ability to engage people in managing their care. Health and social care professionals can engage people by encouraging shared decision making and supporting their ability to self-care [19]. People who are involved in their own health and social care decisions and who are adequately supported to act upon them, feel more respected and empowered, are more likely to adhere to treatment, are more satisfied with their care, and enjoy more positive health outcomes [19,31,34,35,36]. Few responses contained a positive sentiment, while just over a third of the students who participated in this research provided statements that contained negative sentiments in relation to engagement. This could indicate that their understanding and approach to care are not yet person-centred.

When considering the findings more broadly, it is concerning that many students’ responses appeared to align with a more outdated notion that health and social care should be paternalistic, where the health or social care professional makes the decisions for (rather than with) the person [37]. Student responses often reflected a disinterest in answering questions or creating a shared understanding, did not appear to consider the person to be part of the health and/or social care team, and rarely demonstrated a willingness to openly engage in shared decision making. This indicates students are adopting paternalistic models of care which were previously attributed to the ‘hidden curriculum’—the concepts students learn during placements that are not formally or explicitly taught [38,39]. The competitive culture between students in the health professionals’ learning environment, paternalistic-minded educators or placement supervisors, and time pressure were also attributed to students (perhaps unconsciously) adopting this perspective [39,40].

The need to move away from a paternalistic model of care is magnified when considering the needs of people with inadequate health literacy, who are more likely to experience difficulty accessing, understanding, appraising, and using health information in a beneficial way [18]. Almost 60% of Australian adults have inadequate health literacy—a figure that is on par with many other developed countries [41]. Many of the core values needed to provide person-centred care—such as clear communication and cultural capability—are also essential when caring for someone with inadequate health literacy [5,12]. Furthermore, people are able to recognize whether care is paternalistic or person-centred. A recent Australian study explored the health literacy-related barriers and enablers that people face when engaging with health and social care professionals [42]. The authors found that one of the most frequent measures of continued engagement and trust-building was using a person-centred approach, while barriers existed with an absence of person-centredness [42]. A paternalistic-leaning health or social care professional can lead to people delaying or simply not accessing the care they need [42,43]. Additionally, without feeling welcomed to ask questions, people can struggle to make informed health decisions, understand the risks involved with treatment or be in a position to provide informed consent [43,44]. In someone with inadequate health literacy, paternalism can increase the disparity in health care—and therefore health outcomes—when compared to those with adequate health literacy. To more closely adhere to ACSQHC’s recommendation to include person-centred care in health professions’ curricula [5], our findings warrant further exploration to ensure that person-centred care education is explicitly taught and assessed, rather than forming part of the ‘hidden curriculum’. One potential strategy is outlined in the International Community of Practice for Person-centred Practice’s (PcP-ICoP) position statement [45]. The PcP-ICoP have identified a process for developing a health or social care curriculum where person-centredness underpins the entire framework, mirroring its position within recent versions of various professional standards. The key considerations in the PcP-ICoP’s development process include identifying the stakeholders necessary to co-create the person-centred care curriculum, envisioning the needs and varied learning styles of the students, embedding strategies that provide diverse opportunities for assessment, and developing the workplace culture to one with cohesive person-centred values. A rigorous, co-created person-centred care curriculum may help to overcome concerns raised by the present study.

### 4.1. Strengths and Limitations

There are some strengths and limitations of this research, which must be considered. Collecting qualitative data via an online platform meant the researchers could not ask for clarification or further explanation. Therefore, there is the potential that some responses do not reflect the students’ orientation towards person-centred care. However, this approach allowed students the anonymity to participate and give genuine responses in their own words.

Although sentiment analysis software exists, the researchers chose to manually code the data as the software would not detect nuances from a person-centred perspective. However, this did introduce the potential for researcher bias, where personal experience or characteristics could influence the way data were interpreted. Efforts were taken to minimize this effect by using two researchers from different disciplines to independently analyse the data to ensure consistency in interpretation.

The relatively small sample size and restriction of participants to one Australian metropolitan area could limit the transferability of results. However, the curricula explored met national accreditation requirements, so there is potential for the results to be transferable to other similar Australian degrees.

Finally, we were unable to access enrolment numbers for all degrees, so we are unable to comment on the response rate to the survey.

### 4.2. Future Research

Recommendations for future research include increasing the sample size and number of participating universities and supplementing data with interviews or focus groups to provide deeper insight and clarify ambiguous responses. Additionally, few responses aligned with the fourth core value of the person-centred care framework (Collaboration and continuity of care)—future research could incorporate opportunities to gain insight into health and social care professions students’ orientation towards person-centred care within this domain.

## 5. Conclusions

This research provides a valuable and unique insight into health and social care professions students’ orientation towards person-centred care. Health and social care professionals who use a person-centred approach can help people’s access to care, improve satisfaction, and enhance overall health-related outcomes. With the Australian Commission on Safety and Quality in Healthcare’s recommendation to include person-centred care in health professional curricula in mind, sentiment analysis was used to explore health and social care professions students’ orientation towards person-centred care. Almost half of the responses contradicted the core values of person-centred care, indicating that many students may not be orientated towards providing person-centred care. This potential misalignment could continue into their professional careers, the ramifications of which could impact the communities they serve—especially the majority with inadequate health literacy. There is an urgent need to evaluate curricula to ensure they cultivate a person-centred care mindset, possibly using an approach that ensures person-centredness underpins the whole curriculum. This will provide health and social care students more opportunities to develop their person-centred care competencies prior to graduation.

## Figures and Tables

**Table 1:** Demographics of survey participants.

Age	Median 23 Years (IQR 2)	
Gender *n* (%)	Female	61 (68%)
	Male	27 (30%)
	Unknown	2 (2%)
Degree *n* (%)	Pharmacy	31 (34%)
	Podiatric Medicine	26 (29%)
	Social Work and Social Policy	18 (20%)
	Nursing	8 (9%)
	Dental Medicine	7 (8%)

## Data Availability

Not applicable.

## Supplementary Materials

The following supporting information can be downloaded at https://www.mdpi.com/article/10.3390/healthcare11172458/s1, Supplementary Material S1: Table S1. The Checklist for Reporting Results of Internet E-Surveys (CHERRIES). Supplementary Material S2: Survey questions for the current study’s dataset.
