# Supplementary material for: What Do Students’ Questionnaire Responses Tell Us about Their Language around Person-Centred Care? An Exploratory Sentiment Analysis
Source: Healthcare (Basel). 2023 Sep 3;11(17):2458. doi: 10.3390/healthcare11172458 (PMC10487836; doi:10.3390/healthcare11172458)
Supplement: Supplementary file 1 [file healthcare-11-02458-s001.zip › healthcare-2506599-supplementary.pdf]

Supplementary material S1.

Table S1. The Checklist for Reporting Results of Internet E-Surveys (CHERRIES).

Checklist items	Response
**Design**	
**Describe survey design**	Target population: Final year students enrolled in Dental Medicine, Nursing, Pharmacy, Podiatric Medicine, or Social Work and Social Policy.
**Institutional Review Board approval and informed consent process**	
**Institutional approval**	Obtained from The University of Western Australia; approval number is RA/4/20/5960.
**Informed consent**	Consent information was supplied to potential participants as the first survey question. Participants were given information on data storage and security, investigators, potential benefits and risk of participation, and the processes in place to maintain confidentiality. The consent statement required a 'yes' response to access the survey.
**Data protection**	Personal information was collected if participants opted to enter the draw for a gift card; name and contact information were collected in a separate Qualtrics survey. Identifiable information was not able to be traced back to survey responses.
**Development and pre-testing**	
**Development and testing**	The questionnaire was developed by the primary researcher, with extensive review from the research team with expertise in health literacy. After being uploaded to Qualtrics, it was tested for technical functionality before being piloted with five health professions students.
**Recruitment process and description of the sample having access to the questionnaire**	
**Open survey vs closed survey**	Open survey.
**Contact mode**	A QR code and link to the survey were generated through Qualtrics. The survey was disseminated to students enrolled in the abovementioned degrees who met the inclusion criteria. Dissemination occurred via Learning Management Software announcements and email.
**Advertising the survey**	Dissemination occurred via Learning Management Software announcements and email.
**Survey administration**	
**Web/E-mail**	Qualtrics software (web-based).
**Context**	A link to the survey was posted to the Learning Management System for units where eligible students were enrolled.
**Mandatory/Voluntary**	A voluntary and anonymous survey.
**Incentives**	Participants had the opportunity to enter a draw to win an AUD$25 e-gift card.

**Time/date**	The questionnaire was open at different times for each degree, with a convenient time during semester selected by academic staff. Times were selected to avoid clinical/community placements or exam periods which may negatively impact participation.
**Randomization of items or questionnaires**	Not randomized.
**Adaptive questioning**	Not applicable.
**Number of items**	The original questionnaire contained eight items; in this sub-analysis, four items were included.
**Number of screens (pages)**	The original questionnaire used five pages; in this sub-analysis, two pages were included.
**Completeness check**	The consent statement was mandatory to complete. All other questions prompted for a response but it was not forced. Only one question was presented in multiple-choice format (gender); participants could only select one response, and could select "prefer not to say" if they chose.
**Review step**	The back button was purposefully disabled to prevent participants going back to change responses as new information appeared on different pages.
**Response rates**	
**View rate**	Unable to access view rate.
**Participation rate**	142 students visited the first page; 137 agreed to participate. The participation rate was 96%.
**Completion rate**	137 students agreed to participate; 90 completed the survey. The completion rate was 66%.
**Analysis**	
**Handling of incomplete questionnaires**	Only submitted questionnaires were included in analysis. Incomplete questionnaires were excluded.
**Questionnaires submitted with an atypical timestamp**	There were no questionnaires submitted with an atypical timestamp, although all were checked to see if they were submitted too early.
**Statistical correction**	Not applicable.

Supplementary material S2.

Survey questions for the current study's dataset.

1. How old are you? **[open text box]**
2. Which gender do you identify with? **[multiple choice; only one answer permitted]**
    - Male
    - Female
    - Other
    - Prefer not to say

3. Which degree program and year are you currently enrolled in? **[open text box]**

For the rest of this survey, please use the following definition of health literacy when considering your responses: Health literacy is the degree to which individuals have the capacity to obtain, process and understand basic health information and services needed to make appropriate health decisions.

4. What are three signs or behaviours in patients which could indicate inadequate health literacy?

    - **[open text box]**
    - **[open text box]**
    - **[open text box]**
